# Preimplantation genetic testing for concurrent Meckel Syndrome and hereditary breast cancer in a Chinese family harboring a novel *NPHP3* pathogenic variant and a canonical *BRCA2* frameshift variant

**DOI:** 10.1590/1678-4685-GMB-2025-0063

**Published:** 2026-09-07

**Authors:** Yiyuan Zhang, Xianjing Huang, Pingping Qiu, Hong Ji, Lu Ding, Xuemei He, Yingying Shi, Yanru Huang, Ping Li, Libin Mei

**Affiliations:** 1 Xiamen University, School of Medicine, Women and Children’s Hospital, Department of Reproductive Medicine, Xiamen, Fujian, China.; 2 Xiamen University, School of Medicine, Women and Children’s Hospital, Xiamen Key Laboratory of Reproduction and Genetics, Xiamen, Fujian, China.; 3 Xiamen University, School of Medicine, Women and Children’s Hospital, Department of Central Laboratory, Xiamen, Fujian, China.; 4 Xiamen University, School of Public Health, Xiamen, Fujian, China.; 5 Xiamen University, School of Life Sciences, Xiamen, Fujian, China.

**Keywords:** Meckel syndrome, preimplantation genetic testing, NPHP3, BRCA2

## Abstract

Meckel syndrome (MKS) is a lethal autosomal recessive disease with high phenotypic and genetic heterogeneity. Defects in *NPHP3* cause MKS type 7. Herein, we report a case of a Chinese family with a newborn male proband presenting with occipital encephalocele and polycystic kidneys. Whole-exome sequencing was performed on genomic DNA extracted from peripheral blood. Potential variants were assessed for pathogenicity. Two compound heterozygous variants of *NPHP3* (c. 950T>C, p. Phe317Ser, and c.2694-2_2694-1delAG) were identified, which were inherited from both parents, with c.950T>C representing a novel variant. Two *BRCA2* variants (c.5576_5579delTTAA, p. Ile1859Lysfs*3, and c.9357A>C,p. Leu 3119 Phe) were identified, which were inherited from the father. After the proband was diagnosed with MKS7, the couple chose preimplantation genetic testing for monogenic disorders (PGT-M) to simultaneously prevent the transmission of *NPHP3* and *BRCA2* pathogenic variants, leading to a successful pregnancy. Our study expands the *NPHP3* variant spectrum and contributes to the molecular diagnosis and genetic counseling of MKS. This case indicates that PGT-M is a viable option for *NPHP3*-related MKS and *BRCA*-positive patients to avoid transmission while maintaining their families. Successful application of PGT-M provides a potential approach for treating other monogenic diseases.

## Introduction

Meckel syndrome (MKS; OMIM #PS249000) is an extreme form of multiorgan involvement with nephronophthisis (Wolf, 2015), characterized by occipital encephalocele, polydactyly, polycystic kidneys, and hepatic fibrosis (Logan et al., 2010). MKS is inherited in an autosomal recessive manner and is genetically heterogeneous, with multiple pathogenic genes implicated: *MKS1, TMEM216, TMEM67, CEP290, RPGRIP1L, CC2D2A, NPHP3, TCTN2, B9D1, B9D2, TMEM231* (Szymanska et al., 2014)*, CSPP1, TMEM107* (Bondeson et al., 2017)*, TXNDC15*, *CPLANE*, *CEP55*, *TCTN3*, *TMEM237*, *TMEM218* and *TMEM138* (Shaheen et al., 2016; Van De Weghe et al., 2021; Wang et al., 2023; Campobasso et al., 2025; Lo Giudice et al., 2025; Vazquez et al., 2025). Most of these genes are related to ciliary system function; therefore, MKS is also classified as ciliopathy (Simms et al., 2011). MKS is highly genetically heterogeneous, and known pathogenic genes can only explain the etiology in 60% of patients with MKS. MKS also has phenotypic heterogeneity, and its symptoms coincide with those of ciliated nephrotic syndrome (Baala et al., 2007; Delous et al., 2007; Valente et al., 2010), which brings challenges to the differential diagnosis of MKS. MKS is divided into 13 subtypes according to the different pathogenic genes, among which MKS7 (OMIM: 267010) is caused by a homozygous or compound heterozygous variant in *NPHP3*. The *NPHP3* gene is located on chromosome 3q22 and contains 27 exons with a span of 40.5 kb, encoding nephrocystin 3 containing 1330 amino acids, and is highly expressed in the kidneys, especially in renal tubules and glomeruli. Nephrocystin 3 is located in the transition zone between the basal body and cilia axoneme, and affects the development and function of cilia (Omran et al., 2001). Variants in the *NPHP3* gene can lead to Meckel syndrome 7, nephronophthisis 3, and renal-hepatic-pancreatic dysplasia 1.

Breast cancer is one of the most common cancers in women and a rare malignant tumor in men (Fentiman et al., 2006). Breast cancer has an obvious family aggregation, with *BRCA1* (OMIM# 113705) and *BRCA2* (OMIM# 600185) the two most common susceptibility genes for breast cancer (Mahdavi et al., 2019). *BRCA2* is located on chromosome 13q12.3 in human and contains 27 coding exons, a span of 70 kb of genomic DNA and 11.4 kb of transcript, encoding a breast cancer type 2 susceptibility protein containing 3418 amino acids. This protein is located in the nucleus and mainly exists in the breasts, ovaries, testicles, and other reproductive systems. It plays an important role in repairing DNA breaks and is one of the important genes maintaining genomic stability (Roy et al., 2012). Carriers of *BRCA* gene variants have increased risks of breast, ovarian, and prostate cancers.

Here, we describe the case of a Chinese family whose first child was diagnosed with MKS due to two compound heterozygous variants of *NPHP3* accompanied by a *BRCA2* gene variant. PGT-M was successful in helping the mother conceive a healthy child. Our study expands the spectrum of *NPHP3* variants and, for the first time, demonstrates the effectiveness of PGT-M in preventing Meckel Syndrome. Meanwhile, the results confirm that PGT-M is a valuable treatment option for patients with *BRCA* gene variants.

## Subjects and Methods

### Case description

**Figure 1 -:**
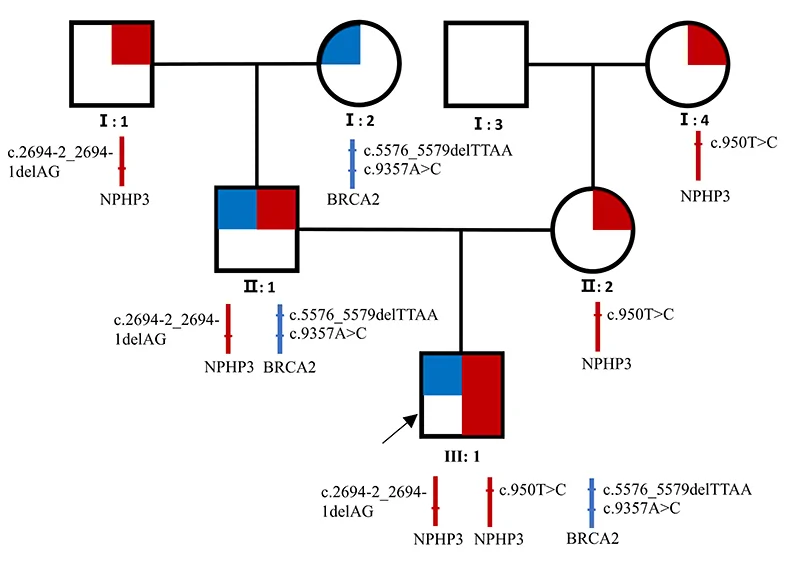
Pedigree of the Chinese family with *NPHP3* and *BRCA2* variants. Red represents the carrying of *NPHP3* gene variant: Ⅰ1 and Ⅱ1 carry the *NPHP3* gene C.2694-2_2694-1del heterozygous variant; Ⅰ4 and Ⅱ2 carry the *NPHP3* gene C.950T >C heterozygous variant; Ⅲ1 carries the complex heterozygous variant of *NPHP3* gene C.2694-2_2694-1delAG and C.950T >C. Blue represents the *BRCA2* gene variant: Ⅰ2, Ⅱ1, and Ⅲ1 carry the *BRCA2* gene C.5576_5579delTTAA and C.9357A >C heterozygous variant. The black arrow indicates the proband (Ⅲ1).

**Figure 2 -:**
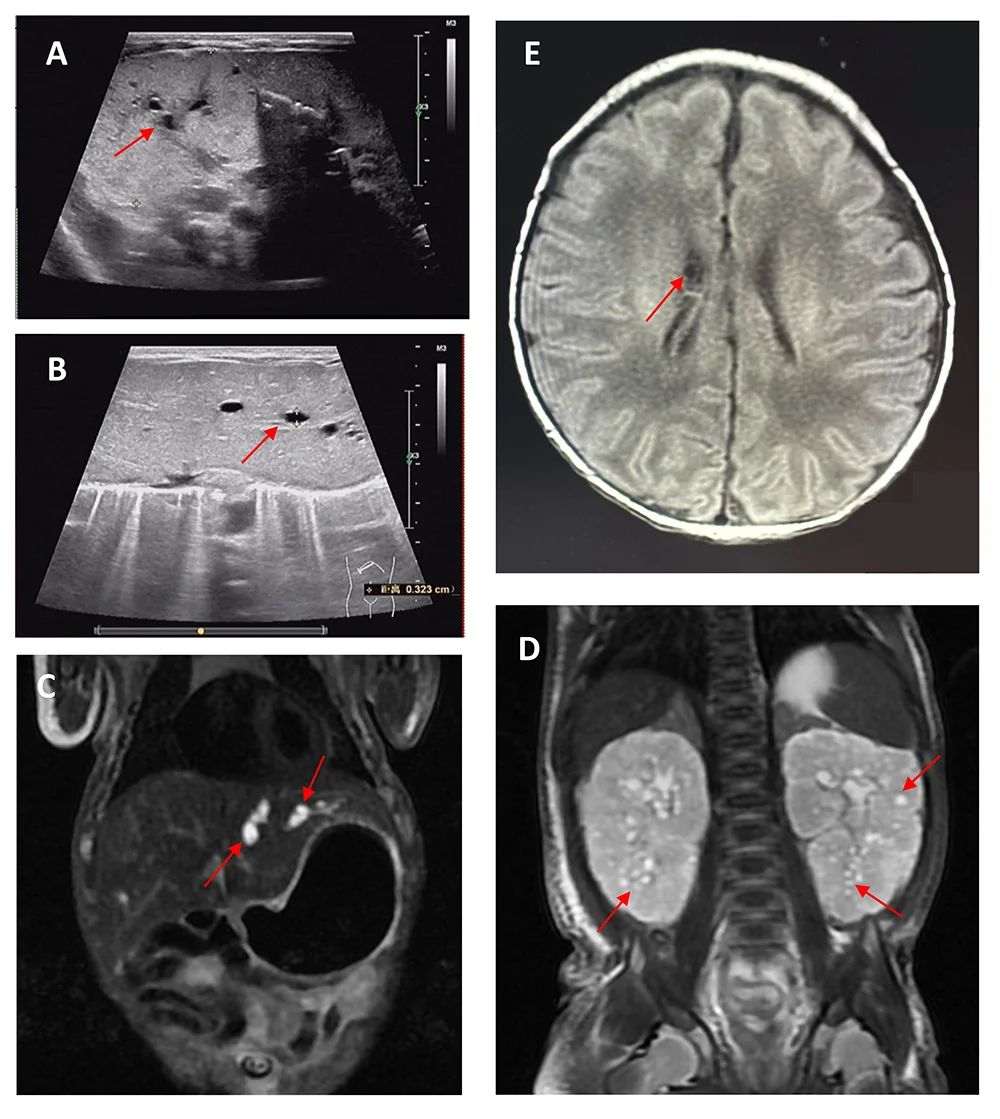
Clinical features of the proband (Ⅲ1). (a) Ultrasound results of the kidney showed that the cortex and medulla were not clearly decomposed, and small cystic echoes were diffused in the parenchyma, indicating an infant polycystic kidney. (b) Cystic dilatation of the small bile duct in the left liver. (c) T2WI fat-suppressed sequence shows multiple cystic and tubular high signal areas in the liver. (d) T2WI fat-suppressed sequence shows enlarged kidneys with multiple diffusely distributed cystic high signal areas in the renal parenchyma. (e) Softening lesion in the anterior part of the right lateral ventricle.

The family in this study is from Fujian, China. The mother (Ⅱ2) was hospitalized for counseling due to a history of adverse pregnancy outcomes and her intention to conceive again. In this previous pregnancy, she delivered a baby boy at term (Figure 1). Ultrasound examination in the second trimester revealed oligoamniotic fluid, slightly enlarged fetal kidneys, enhanced parenchymal echo, and poor corticospinal demarcation. The fetus weighed 3240 g at birth and was hospitalized with fever after birth. A 4 × 4 cm mass was detected on the head. Color Doppler ultrasonography showed infantile polycystic kidneys (left kidney:6.6 × 3.0 × 3.9 cm; right kidney: 6.4 × 3.5 × 3.9 cm) and cystic dilatation of the small intrahepatic bile duct (Figure 2A  and 2B). We observed multiple diffuse sacs in the renal parenchyma, a small amount of fluid accumulated in the pelvic cavity (Figure 2C  and 2D), right subependymal echoless area, and poor brain maturity. Examination showed increased lung texture on both sides, unclear edges, multiple small spots, and small mottled high-density shadows in the inner zone of the lungs on both sides, which was diagnosed as neonatal pneumonia. Cranial magnetic resonance imaging (MRI) showed right lateral ventricular paracalacia (Figure 2E ), and cardiac ultrasonography showed a patent foramen ovale. Chest and abdominal computed tomography (CT) revealed old fractures of the right multiple ribs, abnormal changes in the proximal humerus, and multiple ribs on both sides; a fundus examination revealed hemorrhage at the base of the right eye. To prevent the subsequent transmission of pathogenic variants to the next generation, the couple was counselled and recommended to receive PGT-M treatment. 

### Molecular genetic analyses

Genomic DNA was extracted from peripheral blood leukocytes using a QIAamp blood kit (QIAGEN, Hilden, Germany), following the manufacturer’s protocol. Exons from DNA samples were captured using a BGI MGIEasy V4 chip. High-throughput sequencing was performed using a MGISEQ-2000 sequencer (Wuhan, China). Sequencing reads were aligned to the UCSC human reference genome version hg19 using BWA. GATK and VarScan were used to screen for single-nucleotide polymorphism (SNP) and insertion and deletions in the sequence. The 1000 Genomes, dbSNP, ESP6500, ExAC, and in-house databases were used to select all variant sites with frequencies of < 0.01. Polyphen-2, SIFT, PROVEAN, MutationTaster, and other software were used to predict the pathogenicity of suspected variants, interpreting sequence variants according to the Standards and Guidelines for the Interpretation of Sequence Variants issued by the American College of Medical Genetics and Genomics (ACMG) (Richards et al., 2015) and screened related harmful variants based on the phenotype of the progenitor. The three-dimensional structure of the *NPHP3* protein was predicted using the Alphafold online platform, and its electric potential and structure were visualized and analyzed using PyMOL.

### *In vitro* fertilization and blastomere biopsy

An antagonist scheme was used to promote ovulation, and fertilization was completed by intracytoplasmic sperm microinjection (ICSI). Using Gardner’s blastocyst morphology score method, blastocysts with D5 or D6 ≥ 3BC were selected for trophoblast biopsy. Approximately 5-8 trophoblast cells were transferred to a special collection tube containing 5 μL lysate, and the biopsied blastocysts were vitrified and preserved. Whole-genome amplification (WGA) was performed using multiple annealing and looping-based amplification cycles (MALBAC).

### PGT with haplotype analysis and copy number variation (CNV) analysis 

The WGA products of each embryo were subjected to Sanger sequencing to directly identify the variants. Since only a small number of cells can be used for amplification, it is difficult to avoid allele deletion (ADO). To prevent misdiagnosis, haplotype analysis was conducted using SNP markers. The Illumina Asian Screening Array (ASA) gene chip was used to detect the SNP site on chromosome 3, where the *NPHP3* gene is located, and the SNP site on chromosome 13, where the *BRCA2* gene is located. Within the range of 2 Mb upstream and downstream of the gene, an effective SNP site that could distinguish between high- and low-risk haplotypes was selected for subsequent embryo testing. In addition to the detection of gene variants, CNV analysis was performed using WGA products to prevent embryonic abortion, death, or other problems that may be caused by embryonic chromosomal abnormalities. Deletions or duplications of more than 4 Mb and mosaicism (> 10 Mb) of more than 30% were reported for each embryo.

### Embryo transfer and prenatal diagnosis

The embryo for transfer was selected based on the morphological scores and PGT results. Clinical pregnancy was defined as fetal heartbeat detected by ultrasound 28 days after frozen embryo transfer (FET). To verify the diagnosis of PGT, fetal DNA obtained via amniocentesis at 18 weeks of gestation was subjected to Sanger sequencing, karyotype analysis, and CNV-seq.

This study was approved by the internal ethics committee of Women and Children’s Hospital, Xiamen University (Ethical approval No. KY-2022-090-K02). Written informed consent was obtained from all participants.

## Results

### Genetic analysis results

**Figure 3 -:**
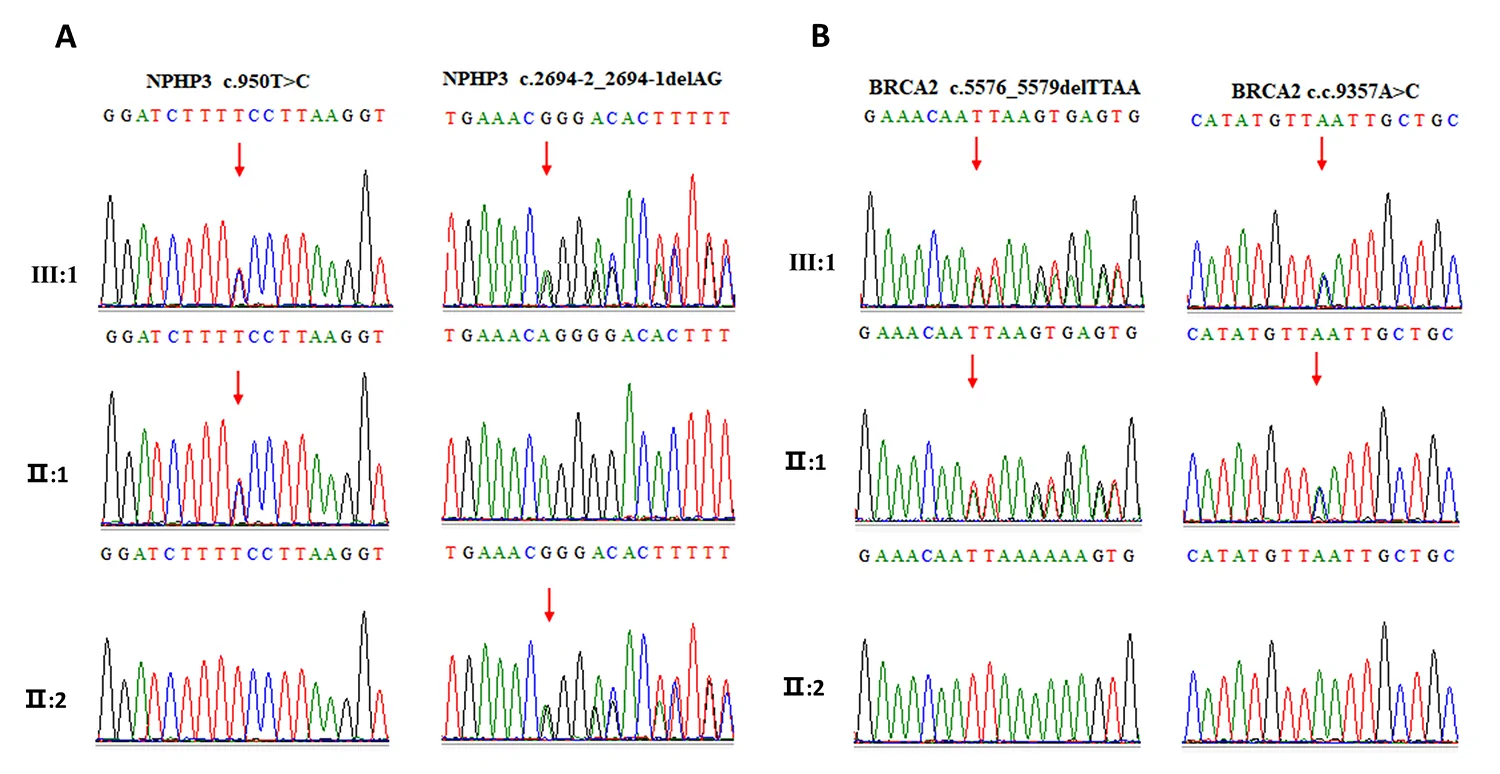
Sanger sequencing of family members Ⅱ1, Ⅱ2, and Ⅲ1 (the proband). The red arrow indicates the variant site. (a) Electropherogram analysis of *NPHP3* in the proband showing compound heterozygous c.950T>C and c.2694-2_2694-1delAG variants of *NPHP3*. The mother (Ⅱ2) carried C.950T>C, while the father (Ⅱ1) carried c.2694-2_2694-1delAG. (b) Electropherogram analysis of *BRCA2* in the proband showing two heterozygous variants, c.5576_5579delTTAA and c.9357A>C, of *BRCA2*, both of which were inherited from the father (Ⅱ1). The c. 950T>C variant is a missense variant in exon 5 of *NPHP3* that results in a phenylalanine substitution for serine at amino acid 317 (p.F317S).

Whole-exome sequencing analysis identified two compound heterozygous variants in *NPHP3* (NM_153240.4), c.950T>C (p. Phe317Ser) and c.2694-2_2694-1delAG, in the proband (Ⅲ1). Sanger sequencing showed that the c.950T>C variant was maternally inherited, while the c.2694-2_2694-1delAG variant was paternally inherited, indicating complete segregation of the variants with the disease phenotype (Figure 3A and 3B). 

**Figure 4 -:**
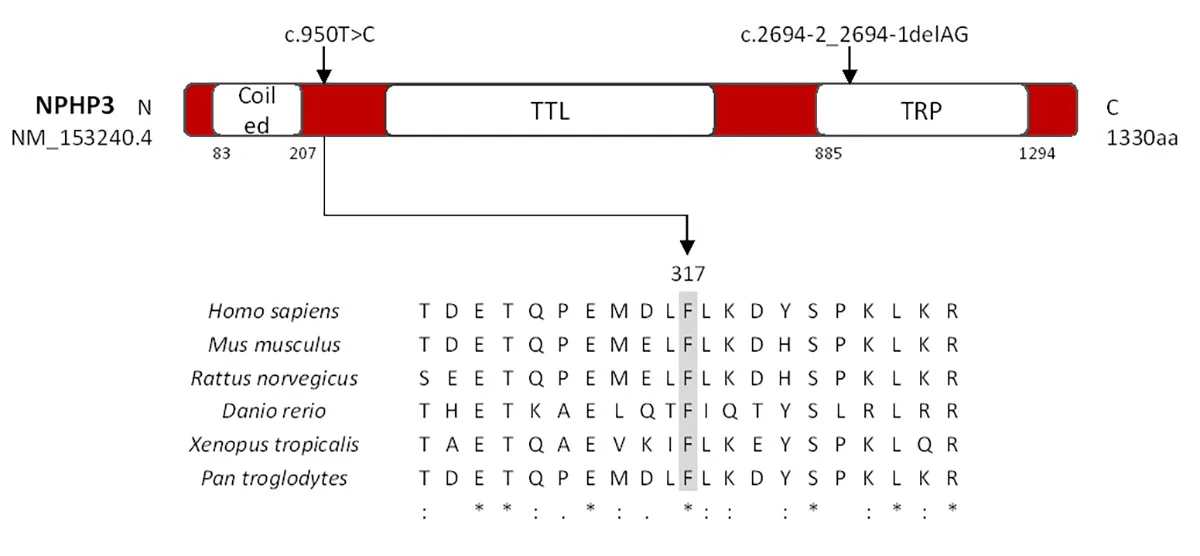
Structures of the *NPHP3* gene, along with prediction of the conservation of c.950T>C. The red structure represents the *NPHP3* gene, a.a.83-207 coiled coil (CC) domain, a tubulin-tyrosine ligase (TTL) domain, and a.a.885-1294 a tetratrico peptide repeat (TPR) domain. Variant c.2694-2_2694-1delAG occurs in the intron 19 region, which may affect exon20 expression in the TRP region. c. 950T>C missense variant is located in the intervening sequences between the CC domain and TTL domain. And the c. 950T>C (p.F317S) is conservation of in multiple species, which were highly conservative.

**Figure 5 -:**
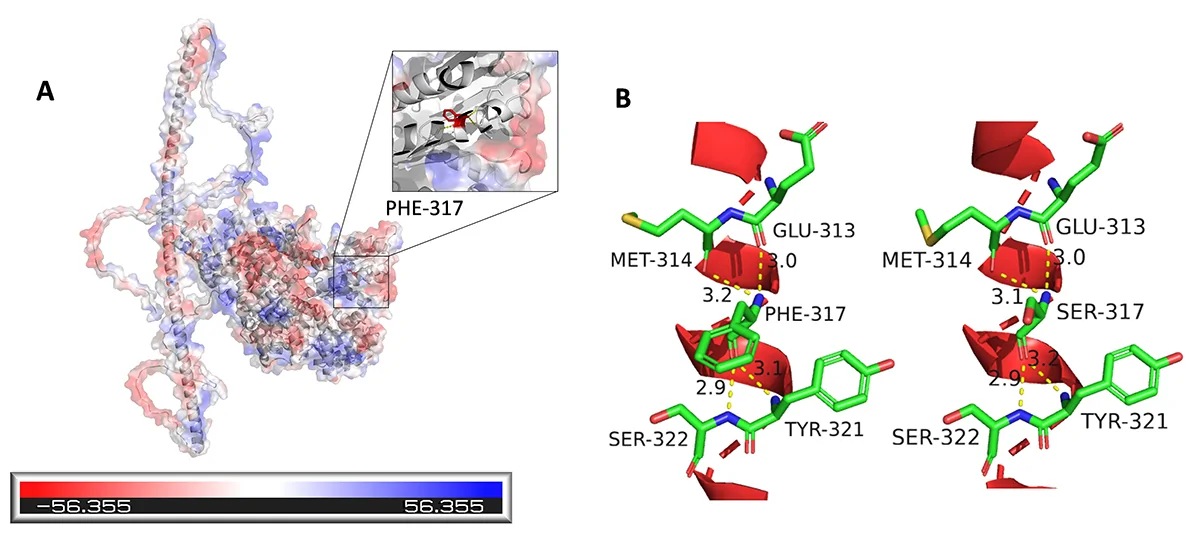
Schematic diagram of the *NPHP3* protein and mutein. (a) Schematic diagram of the sequence structure of the *NPHP3* protein predicted by Alphafold. (b) Structures of the *NPHP3* peptides containing either 317Phe or 317Ser.

Cluster X analysis showed that the phenylalanine variant site was highly conserved among different species (Figure 4). Pathogenesis prediction programs indicated that c. 950T>C was “potentially destructive” (PolyPhen-2; score: 0.998, sensitivity: 0.69, specificity: 0.90), “destructive” (SIFT; score: 0.002), and “neutral” (PROVEAN; score: -2.02). The structure of the wild-type protein and its electrostatic potential are shown (Figure 5A ). Missense variants occur in regions enriched with positive charges. Comparison of the *NPHP3* protein structures containing 317F and 317S revealed that the position of the amino acid at residue 317 and its interactions with neighboring atoms changed, leading to a slight conformational alteration in the protein structure (Figure 5B ). This variant has not been previously reported and was not found in ClinVar, HGMD, ExAC, ESP6500, dbSNP, or any other single-nucleotide polymorphism databases. According to the ACMG guidelines (Richards et al., 2015), the variant c. 950T>C was classified as a variant of uncertain significance (VUS) (PM2/PM3_Supporting/PP3).

Variant c.2694-2_2694-1delAG is a splicing variant that results in deletion of the first and second base AG of the splice site in exon 19 of *NPHP3*. This variant was first reported in a Turkish family with Meckel-like syndrome and consanguineous marriage. According to the ACMG guidelines (Richards et al., 2015), the variant c.2694-2_2694-1delAG was classified as pathogenic (PVS1/PM2/PM3/PP1_Moderate).

In addition, the proband was found to have two heterozygous variants, c.5576_5579delTTAA (p. Ile1859Lysfs*3) and c.9357A>C (p. Leu 3119 Phe), in the *BRCA2* gene (NM_000059.3), both of which were inherited from the father. c.5576_5579delTTAA is a frameshift variant located in exon 11 of *BRCA2* gene, and is predicted to result in a frameshift at codon 1859 with substitution of a lysine for an isoleucine and introduction of a putative stop codon 3 amino acids downstream in the translated protein (p. I1859Kfs*3); it is classified as pathogenic variant according to the ACMG. c.9357A>C is a missense variant located in exon 25 of the *BRCA2* gene, resulting in the substitution of 3119 amino acid leucine by phenylalanine (p. L3119F); it is classified as a VUS according to the ACMG.

No other significant variants were identified in the WES analysis.

### Embryo preparation and genetic testing

Ten embryos were fertilized by ICSI, of which seven were suitable for biopsy. We successfully amplified blastocyst DNA using MALBAC. The CNV results showed that six embryos (E1, E2, E3, E4, E5, and E6) had normal karyotypes, whereas only one (E7) had an abnormal karyotype. Sanger sequencing revealed that E1 carried *NPHP3* c.2694-2_2694-1delAG and *BRCA2* c.5576_5579delTTAA variants, E2 carried neither *NPHP3* nor *BRCA2* pathogenic variant, E3 carried *NPHP3* c.2694-2_2694-1delAG and c.950T>C variants, E4 carried *NPHP3* c.950T>C variant, E5 carried *BRCA2* c.5576_5579delTTAA variant, and E6 carried *NPHP3* c.2694-2_2694-1delAG variant. 

To verify the Sanger sequencing results, we selected SNPs that could be used to distinguish between high- and low-risk haplotypes to infer the presence of chromosomes in embryos. SNP-based haplotyping showed that E1 inherited the paternal high-risk chromosome carrying the *NPHP3*:c.2694-2_2694-1delAG variant and paternal high-risk chromosome carrying the *BRCA2*:c.5576_5579delTTAA variant; E3 inherited both the maternal high-risk chromosome carrying the *NPHP3*:c.950T>C variant and paternal high-risk chromosome carrying the *NPHP3*:c.2694-2_2694-1delAG variant; E4 inherited the maternal high-risk chromosome carrying the *NPHP3*:c.950T>C variant; E5 inherited the paternal high-risk chromosome carrying the BRCA2:c.5576_5579delTTAA variant; E6 inherited the maternal high-risk chromosome carrying the NPHP3:c.950T>C variant. This was consistent with the Sanger sequencing results (Table 1).

**Table 1 -:** Genetic screening results of embryos.

Embryo	Biopsy time	Gardner grade^a^	Copy number variations	SNP haplotyping^b^	Sanger sequencing
E1	D5	4BB	46, XN	F-Hap A/M-Hap B F-Hap C	NPHP3:c.2694-2_2694-1delAG BRCA2:c.5576_5579delTTAA
E2	D5	4BC	46, XN	F-Hap B/M-Hap B F-Hap D	normal
E3	D5	4BC	46, XN	F-Hap A/M-Hap A F-Hap D	NPHP3:c.2694-2_2694-1delAG NPHP3:c.950T>C
E4	D5	4BC	46, XN	F-Hap B/M-Hap A F-Hap D	NPHP3:c.950T>C
E5	D5	4BC	46, XN	F-Hap B/M-Hap B F-Hap C	BRCA2:c.5576_5579delTTAA
E6	D5	4BC	46, XN	F-Hap A/M-Hap B F-Hap D	NPHP3:c.2694-2_2694-1delAG
E7	D6	4BC	+21	F-Hap B/M-Hap A F-Hap D	NPHP3:c.950T>C

^a^ The embryo quality was assessed based on morphological characteristics following the Gardner grading system (https://www.lifewhisperer.com/wp-content/uploads/2022/03/Gardner-grading-guide.pdf). ^b^F-Hap A: haplotype A of the father,NPHP3,c.2694-2_2694-1delAG; F-Hap B: haplotype B of the father, NPHP3,wild- type; M-Hap A: haplotype A of the mother,NPHP3, c.950T>C; M-Hap B: haplotype B of the mother, NPHP3,wild- type. F-Hap C: haplotype C of the father, wild- type, BRCA2,c.5576_5579delTTAA, F-Hap D:haplotype D of the father, wild- type, BRCA2,wild- type.

### Prenatal diagnosis and pregnancy outcome

Based on embryo selection principles, the euploid embryo E2 that did not carry *NPHP3* or *BRCA2* variants was transferred into the uterus. The serum human chorionic gonadotropin level 13 days after transplantation was 8283 mIU/ml, and ultrasound examination at 28 days indicated an intrauterine single birth. The prenatal diagnostic results were consistent with those of PGT-M. A healthy male infant was born at the 38th week of gestation.

## Discussion

Meckel syndrome (MKS) is an autosomal recessive ciliopathy and represents the most severe, multi-organ form of nephronophthisis (NPHP)-associated kidney disease. MKS is a rare condition with a global incidence of from 1/13,250 to 1/140,000 live births (Parelkar et al., 2013). MKS is characterized by marked genetic and phenotypic heterogeneity, which often overlaps with other ciliopathies and syndromic disorders, thereby complicating clinical diagnosis. The diagnosis of MKS is primarily established based on the classic triad of occipital encephalocele, cystic kidneys, and fibrotic changes to the liver. (Carey et al., 1980). In this study, we report the case of a Chinese male fetus presenting with cystic kidney disease, occipital encephalocele, hepatosplenomegaly, and pulmonary abnormalities. We identified two compound heterozygous variants in *NPHP3* in a patient presenting with severe clinical manifestations of MKS.

*NPHP3* was first linked to kidney disease in 2000 in a study of a Venezuelan family with adolescent nephrotic syndrome (Fiskerstrand et al., 2010). Subsequent studies have demonstrated that variants in *NPHP3* are associated with a phenotype spectrum ranging from severe MKS to milder ciliopathies, including nephronophthisis 3, renal-hepatic-pancreatic dysplasia 1, and Meckel syndrome 7. These conditions are characterized by multisystem involvement, including ocular, hepatic, limb (polydactyly), cardiac, and congenital kidney and urinary tract (CAKUT) abnormalities, and are classified as NPHP-related ciliopathies (NPHP-RC) (Halbritter et al., 2013).

Nephrocystin 3 is localized at the transition zone of the primary cilium. The bridging component consists of Nephrocystin-2-3-9 and is expressed along the entire axoneme (Wolf, 2015). Nephrocystin 3 consists of a coiled coil (CC) domain, a tubulin-tyrosine ligase (TTL) domain, and tetratricopeptide repeat (TPR) domain. Nephrocystin 3 interacts with inversin to inhibit the canonical Wnt pathway (Molinari et al., 2018). Most proteins of the NPHP gene family are localized in the cilia and variants can lead to ciliary nephropathies. Studies have shown that NPHP proteins interact to form functional modules (Bergmann et al., 2008). Nephrocystin 3, Nephrocystin 2, and Nephrocystin 9 form a module located in the inversin compartment of the cilia, connecting the ciliary axoneme to the cilia basal body. Studies have suggested that loss of this module is associated with infantile nephronophthisis and cardiac defects (Wolf, 2015). The proband exhibited a phenotype consistent with infantile nephronophthisis, with disease onset at birth. The proband was ultimately fatal. Although the proband did not have any congenital heart defects, a patent foramen ovale (PFO) was observed. Similar cases of renal disease accompanied by PFO have been previously reported in the literature (Halbritter et al., 2013). In mouse models, a missense variant in Nphp3 leads to a cystic kidney disease phenotype, and the complete loss of Nphp3 gene function results in situs inversus, congenital heart defects, and embryonic lethality (Olbrich et al., 2003). Similarly, variant in the Nphp3 gene leads to hydrocephalus and nephrogenic cysts in zebrafish embryos (Molinari *et al.*, 2018).

The c.950T>C variant is located in the linker region between the CC and TTL domains, where CC is essential for localization (Nakata et al., 2012), and the TTL domain is involved in detyrosination and microtubule functions (Olbrich et al., 2003, Szyk et al., 2011). Structural modeling suggested that the c.950T>C variant disrupts interactions between Phe 317 and Met 314, as well as between Phe 317 and Tyr 321, potentially leading to conformational alterations of the protein (Figure 5A  and 5B). However, the precise pathogenic mechanism of this variant remains to be elucidated. The c.2694-2_2694-1delAG variant is located in intron 19 and was first reported in homozygous form by Bergmann. (Bergmann et al., 2008) in a family with an MKS-like phenotype. This variant introduces a premature stop codon into intron 19, resulting in the production of a truncated protein. Torunn Fiskerstrand et al. (Fiskerstrand et al., 2010) found that the c.2694-2_2694-1delAG variant can lead to the alternative splicing of exon 20, producing a transcript lacking exon 20. Exon 20 is located within the TRP repeat domain and mediates protein-protein interactions. Deletion of this region may cause ciliary dysfunction, leading to ciliopathies, with MKS being a classic example. Halbritter et al. (Halbritter et al., 2013) reported several cases of compound heterozygous variants, including c.2694-2_2694-1delAG, combined with different missense or nonsense variants, such as c.518A>G, c.1928C>T, c.2369T>C, and c.3020T>G. These patients exhibit MKS phenotypes similar to those of the proband in this study. Collectively, these findings support NPHP3 as the causative gene in this patient.

The correlation between genotypes and phenotypes of *NPHP3* variants remains unclear. Some studies have suggested that *NPHP3* missense variants are more likely to result in isolated NPHP, whereas truncated variants are associated with NPHP-RC involving multiple organs (Chaki et al., 2011). In contrast, other studies indicate that the severity of *NPHP3* variants does not consistently predict the severity of renal phenotypes. (Meng et al., 2017). Hoefele et al. (2007) demonstrated that oligogenic inheritance can influence the phenotypic severity of *NPHP3*, suggesting that carrying a third pathogenic variant may result in a more severe disease phenotype in NPHP-RCs. Additionally, gene modification affects the severity of the NPHP phenotype (Chaki *et al.*, 2011; Meng *et al.*, 2017).

In this study, the proband carried two heterozygous variants in *BRCA2*, c.5576_5579delTTAA (p. Ile1859Lysfs*3) and c.9357A>C (p. Leu3119Phe), which were inherited from the father and paternal grandmother. The grandmother had undergone mastectomy for breast cancer. The c.5576_5579delTTAA variant is located in exon 11 of *BRCA2*, which is the largest exon of the gene and the most important and represents a major hotspot for pathogenic variants in breast cancer patients (El Saghir et al., 2015; Kim et al., 2016). Exon 11 contains eight key functional BRC repeat domains and is the site of Rad51 binding (Cheng et al., 2018; Andreassen et al., 2021). The variant is located in BRC repeat 6, a region previously reported to harbor pathogenic variants. The c.9357A >C variant is a missense variant located in exon 26 of the *BRCA2* gene and has been classified by the ACMG as a VUS. Most pathogenic variants in *BRCA1* and *BRCA2* are small insertions or deletions that result in nonsense, frameshift, or splice site variants, all of which lead to premature protein truncation. Approximately 2% of pathogenic *BRCA1* variants are caused by missense variants, most of which are classified as VUS (Mazoyer, 2005).

*BRCA2* gene defects are also strongly associated with male breast cancer and increase the risk of testicular and prostate cancers, potentially leading to spermatogenic dysfunction and an increased risk of male infertility. Evidence suggests that *BRCA* variant carriers remain cancer-free during their reproductive years; however, *BRCA2* variants can lead to earlier onset of associated cancers. In this study, the proband’s father carried a *BRCA2* variant, but exhibited no symptoms. However, this does not imply that the variants are nonpathogenic or that PGT is unnecessary. *BRCA* gene variants do not inevitably result in disease manifestations, and the prevalence of *BRCA* variants is influenced by environmental factors. Nonetheless, during genetic counseling, couples are informed of the substantial risks associated with *BRCA* variants to prevent the inheritance of pathogenic variants, sparing future generations from the associated disease risks (Dong et al., 2021). Following genetic counseling, the couple opted for PGT to prevent the transmission of pathogenic variants, resulting in a wild-type euploid embryo without both the pathogenic variants and a successful pregnancy.

In this study, we conducted a genetic diagnosis of a child with MKS and identified a causative variant, which included a previously unreported *NPHP3* gene variant, c.950T>C. This study expands the spectrum of *NPHP3* variants and provides the first evidence that PGT-M can effectively prevent the transmission of Meckel syndrome. Moreover, PGT-M offers a viable reproductive option for *BRCA* variant carriers to avoid transmitting pathogenic variants to future generations.

## Data Availability

The raw data supporting the conclusions of this article will be made available by the authors on request.
